# Lidocaine clearance as pharmacokinetic parameter of metabolic hepatic activity in patients with impaired liver

**DOI:** 10.5937/jomb0-38952

**Published:** 2023-03-15

**Authors:** Marija Jovanović, Milena Kovačević, Sandra Vezmar-Kovačević, Ivan Palibrk, Jasna Bjelanović, Branislava Miljković, Katarina Vučićević

**Affiliations:** 1 University of Belgrade, Faculty of Pharmacy, Department of Pharmacokinetics and Clinical Pharmacy, Belgrade; 2 University Clinical Centre of Serbia, Department for Anesthesia and Reanimation, Section at Clinic for Digestive Surgery, Belgrade; 3 University Clinical Centre of Serbia, Center for Medical Biochemistry, Belgrade

**Keywords:** liver failure, lidocaine, pharmacokinetics, insuficijencija jetre, lidokain, farmakokinetika

## Abstract

**Background:**

The study aimed to estimate lidocaine (LID) pharmacokinetic parameter values in patients with impaired liver function, level of correlation between the pharmacokinetic parameters and Child-Pugh class and change in pharmacokinetic parameters after liver tumor resection compared to the preoperative value.

**Methods:**

Patients with impaired liver function were subject to the LID test 1 day prior to, 3 and 7 days after the intervention. LID was administered in single i.v. dose of 1 mg/kg. Blood samples were collected at 15, 30 and 90 minutes after drug administration. Non-compartmental analysis was applied for calculating the pharmacokinetic parameters.

**Results:**

The study included 17 patients with the diagnosis of cirrhosis and 41 patients with liver tumor. In both groups of patients, the values of the coefficients of correlation show the best correlation between clearance (CL) and Child-Pugh score (-0.693, p<0.005) over other pharmacokinetic parameters. The results indicate worsening hepatic function on 3rd day after operation in comparison to the values of LID CL prior to operation (mean LID CL for patients with Child-Pugh class A are 25.91 L/h, 41.59 L/h, respectively; while for B class are 16.89 L/h, 22.65 L/h, respectively). On day 7th, the values of LID CL (mean value for patients with Child-Pugh class A and B are 40.98 L/h and 21.46 L/h, respectively) are increased in comparison to 3rd day after.

**Conclusions:**

LID pharmacokinetic parameters consequently changed according to the severity of liver impairment, assessed by Child-Pugh score. Values of LID CL and volume of distribution (Vd) coupled with standard biochemical parameters may be used for preoperative assessment of liver function and monitoring of its postoperative recovery.

## Abstract (sr)

**Uvod:**

Cilj studije bila je procena vrednosti farmakokinetičkih parametara lidokaina (LID) kod pacijenata sa oštećenom funkcijom jetre, stepena korelacije između farmakokinetičkih parametara i Child-Pugh klase i promene farmakokinetičkih parametara posle resekcije tumora jetre u odnosu na preoperativnu vrednost.

**Metode:**

Pacijenti sa oštećenom funkcijom jetre bili su podvrgnuti LID testu 1 dan pre, 3. i 7. dana nakon intervencije. LID je primenjen u pojedinačnoj i.v. dozi od 1 mg/kg. Uzorci krvi su sakupljeni 15, 30 i 90 minuta nakon primene leka. Za izračunavanje farmakokinetičkih parametara primenjena je neprostorna analiza.

**Rezultati:**

Studijom je obuhvaćeno 17 pacijenata sa dijagnozom ciroze i 41 pacijent sa tumorom jetre. Kod obe grupe pacijenata, vrednosti koeficijenata korelacije pokazuju najbolju korelaciju između klirensa LID (CL) i Child-Pugh skora (-0,693, p<0,005) u odnosu na ostale farmakokinetičke parametre. Rezultati ukazuju na pogoršanje funkcije jetre 3. dana nakon operacije u poređenju sa vrednostima LID CL pre operacije (srednje vrednosti LID CL kod pacijenata Child-Pugh grupe A iznosile su 25,91 L/h, 41,59 L/h, respektivno; dok su kod pacijenata u klasi B iznosile 16,89 L/h, 22,65 L/h, respektivno). Sedmog dana vrednosti LID CL (srednja vrednost u Child-Pugh grupi A i B iznosile su 40,98 L/h i 21,46 L/h, respektivno) bile su veće u odnosu na 3. dan posle hirurške intervencije.

**Zaključak:**

Farmakokinetički parametri LID se razlikuju u zavisnosti od težine oštećenja jetre, procenjenih Child-Pugh skorom. Vrednosti farmakokinetičkih parametara LID u kombinaciji sa standardnim biohemijskim parametrima mogu se koristiti za preoperativnu procenu funkcije jetre i praćenje njenog postoperativnog oporavka.

## Introduction

The assessment of liver disease severity and prediction of preoperative hepatic function, pre-transplantation status of recipients, and post-transplantation survival of patients is vital in clinical practice. While cirrhosis or end-stage liver disease denotes impaired liver function caused by fibrosis due to longterm liver damage, liver cancer is associated with abnormal uncontrolled growth in the liver. However, cirrhosis is also risk factor for primary liver cancer, such as hepatocellular carcinoma, which can develop at any stage of cirrhosis. Hence, the degree of underlying liver cirrhosis is important to consider in treatment decisions and prognosis of patients [1]. Using biochemical parameters in liver failure assessment is not a satisfactory overall representation of the functional status of the organ [2]. The most frequently used scale based on biochemical parameters (albumin, bilirubin, prothrombin time), and clinical signs (presence of ascites, encephalopathy) is Child-Pugh classification [1] [3]. Different dynamic liver tests using probe substances (e.g. indocyanine green, galactose, cholate, aminopurine, methacetin, caffeine, and lidocaine) might be used in order to predict hepatic metabolic function, since the liver volume could overestimate liver function [4] [5] [6] [7]. Lidocaine (LID) undergoes extensive hepatic biotransformation (around 97%) via cytochrome P (CYP) 3A4 and 1A2 to monoethylglycinexylidide (MEGX) and 3-hydroxylidocaine [8] [9] [10]. Being a high extraction drug, LID metabolism is dependent on hepatic blood flow, and different hepatic diseases may have different effect on primary pharmacokinetic parameters such as clearance (CL) or volume of distribution (Vd), and consequently on half-life (t_1/2_). According to the literature data, LID average t_1/2_ values are about 100 min, while CL ranges from 10 to 20 mL/minxkg, in the healthy subjects [10]. In the clinical practice, single measurement of MEGX after injection of LID test dose is extensively used as an indicator of liver function and metabolic reserve [10] [11] [12] [13] [14]. Formation rates of MEGX decrease with increasing severity of liver disease, and correlation of MEGX single point concentration with Child-Pugh class has been shown [15] [16]. Since the optimal time for blood sampling varies between studies (15, 30 or 60 minutes), the results of previous studies are inconsistent about the use of MEGX concentration measurement at a fixed time point after LID administration as an indicator of metabolic hepatic activity [11] [12] [13] [17] [18]. Single MEGX concentration is static indicator of liver function characterized by wide interindividual variability, whereas pharmacokinetic parameters based on LID time-depending levels might be better predictors of hepatic function [7] [19] [20]. It was found that elimination t_1/2_ of LID is more closely related to the Child-Pugh's staging of liver dysfunction than 15-minute MEGX concentration [21]. Data on LID pharmacokinetics, and the correlation with Child-Pugh class are scarce and the published articles are mainly focused on the cirrhotic patients [21] [22]. Consequently, we performed a study that aimed the estimation of the pharmacokinetic characteristics of LID in patients with liver cirrhosis and tumor, finding the level of correlation between the pharmacokinetic parameters and Child-Pugh class, and to assess change in pharmacokinetic parameters after liver tumor resection compared to the preoperative value.

## Materials and methods

### Patients

The prospective study was conducted at the Department for Anesthesia and Reanimation, Section at Clinic for Digestive Surgery, University Clinical Centre of Serbia. The study included patients diagnosed with different liver impairments, aged 18 years or older. According to the pathophysiology of the hepatic diseases, patients were divided into two groups: patients with cirrhosis, and patients with tumors. Diagnosis was based on clinical, biochemical, endoscopic, ultrasonographic evaluation, contrast enhanced multislice computerized tomography (MSCT) or magnetic resonance imaging (MRI) scan and liver biopsy, where indicated. The inclusion criteria for patients with cirrhosis were terminal stage of liver failure and registration on the waiting list for liver transplantation. Patients with a tumor could be included in the research if they had primary or metastatic tumor in the liver, where a liver resectability was expected up to the resection margin. The study excluded patients with extrahepatic spread of malignant disease, inoperability determined at surgery, concomitant therapy that significantly influence LID pharmacokinetics, severe cardiac disease or central nervous system disorders which could be worsened by i.v. LID administration, underlying health problems which could affect blood flow, LID allergy, and the absence of the written informed consent.

The study was approved by the local ethic committee, conducted in accordance with the Declaration of Helsinki, and all patients provided informed consent.

### Lidocaine administration, blood sampling and biochemical analysis

The following serum biochemical parameters were measured in all patients: total bilirubin (BIL), aspartate aminotransferase (AST), alanine aminotransferase (ALT), gamma glutamyl transpeptidase (GGT), alkaline phosphatase (ALP), albumin (ALB), and presence of ascites, encephalopathy, international normalized ratio (INR) were recorded. These data were used to calculate Child-Pugh score, and consequently Child-Pugh class. Biochemical parameters: BIL, AST, ALT, GGT, ALP, ALB were measured by spectrophotometry (Olympus AU400, OLYMPUS). Hemo stasis parameters: prothrombin time (PT) and INR were measured by photo-optical coagulometry (ACL9000, Instru mentation Labo ratory). Patients were subjects to LID test 1 day prior to, 3 and 7 days after the surgical intervention. LID was administered* i.v.* in a single dose of 1 mg/kg over 2 minutes. Preoperative sampling time was 15, 30 and 90 minutes after LID administration. Blood samples, from contralateral arm, were collected after 15, 30 and 90 minutes, and in 6 patients after 15, 30, 90 and 120 minutes.

### Bioanalytical and pharmacokinetic analysis

LID levels were measured by TDx fluorescence polarization immunoassay (FPIA) technique using commercially available analysis kit (Abbott Laboratories, Diagnostic Division, Chicago, Illinois, USA).

Based on measured LID levels, individual concentration vs. time profile was constructed. Individual pharmacokinetic parameters were calculated using non-compartmental analysis and they included: first order rate constant associated with terminal (log-linear) portion of concentration-time curve (Λ_z_), terminal t_1/2_, total CL, and Vd.

### Statistical analysis

Descriptive and statistical analysis was performed using PASW Statistics^®^ (version 22, Chicago, Illinois, USA). In order to determine the degree of association between Child-Pugh class and t_1/2_, CL, or Vd of LID, correlation analysis was performed. Both parametric and nonparametric methods were considered according to the normal distribution of the data, and specific coefficients of correlation were calculated. The total sample size was calculated to 31 patients (95% confidence level, 5% margin of error, 2% the total population proportion for cirrhosis and liver cancer). The cirrhosis group size was estimated to minimum of 10-13 patients (0.6% prevalence in Serbia; 0.83% median prevalence in Europe), whereas the tumor group size was estimated to minimum of 16 patients (up to 1% prevalence in Europe) [23] [24].

## Results

The study included 17 patients with the diagnosis of cirrhosis where patients' mean age was 47 years, and 41 patients with liver tumor aged in average 60 years. The characteristics of the patients included in this study are presented in Table 1. No statistical difference was observed between the studied groups in their demographic characteristics, except that patients with cirrhosis in average were younger than patients with tumor.

**Table 1:** Patients’ characteristics.

		patients with<br>cirrhosis	patients<br>with tumor	p-value
Number of patients		17	41	
male		9 (52.94%)	28 (68.29%)	0.268
Body weight [kg]		77.85 ± 12.56	73.54 ± 16.29	0.332
Age [years]		47.31 ± 15.72	59.90 ± 10.05	0.001
Positive smoking status		8 (47.06%)	25 (60.89%)	0.330
Alcohol consumption		10 (58.82%)	26 (63.41%)	0.743
Child-Pugh class	A	7 (41.18%)	34 (82.93%)	0.001
	B	10 (58.82)	7 (17.07%)	

**Figure 1:**
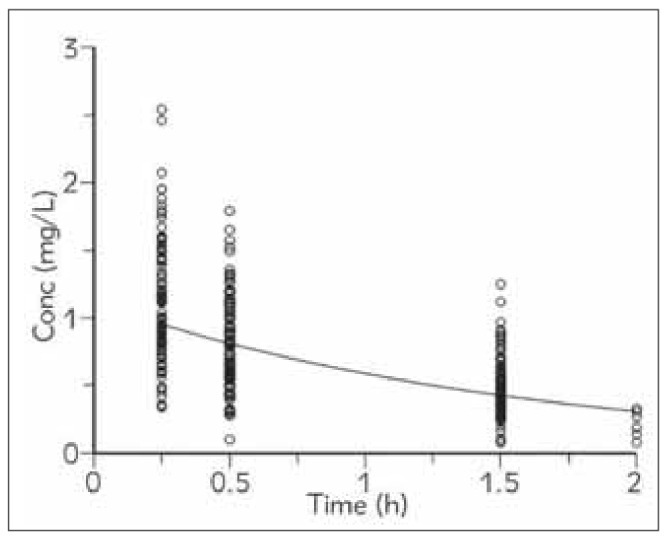
Mean lidocaine (LID) concentration profile with individual measured levels.

Mean concentration vs. time LID profile with individual measured levels is presented on Figure 1. Based on individual concentration vs. time profiles, individual and correspondently mean pharmacokinetic parameters (λ_z_, t_1/2_, CL, Vd) were calculated using non-compartmental analysis.

**Figure 2:**
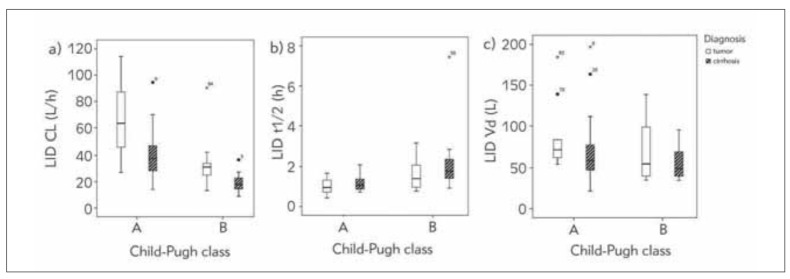
Boxplots of lidocaine (LID): a) clearance (CL), b) half-life (t_1/2_), c) volume of distribution (Vd) values in relation to Child-Pugh class in patients with tumor or cirrhosis.

Further analysis showed that LID pharmacokinetic parameters were altered with a severity impairment of liver function, assessed by Child-Pugh class; thus CL and λ_z_ decreased, while t_1/2_ prolonged both in patients with diagnosed tumors and cirrhosis (Figure 2a, Figure 2b). Figure 2c represents changes of LID Vd values in function of Child-Pugh class in patients with tumors and cirrhosis.

Since data did not follow normal distribution, nonparametric correlation tests were used to assess the association between pharmacokinetic parameters of LID and Child-Pugh score. The results of the analysis are given in Table 2. The results indicate better correlation when the analysis was performed in relation to patients' diagnosis (Table 2). In both groups of patients, the values of the coefficients of correlation show the best correlation between CL and Child-Pugh score over other pharmacokinetic parameters; where coefficient of correlation between CL and Child-Pugh score was -0.693 and -0.543 in patients with cirrhosis and with tumor, respectively (Table 2).

**Table 2:** Coefficients of correlation between pharmacokinetic parameters of lidocaine (LID) and Child-Pugh score. ^*^significant correlation at the 0.05 level<br>^**^significant correlation at the 0.001 level

Lidocaine (LID)<br>pharmacokinetic <br>parameters	Coefficient of correlation<br>with Child-Pugh score		
	all<br>patients	patients<br>with cirrhosis	patients<br>with tumor
clearance (CL)	- 0.434^*^	- 0.693^**^	- 0.543^**^
distribution (Vd)	- 0.101	- 0.250	- 0.132
half-life (t_1/2_)	0.350^*^	0.346	0.465^**^

**Figure 3:**
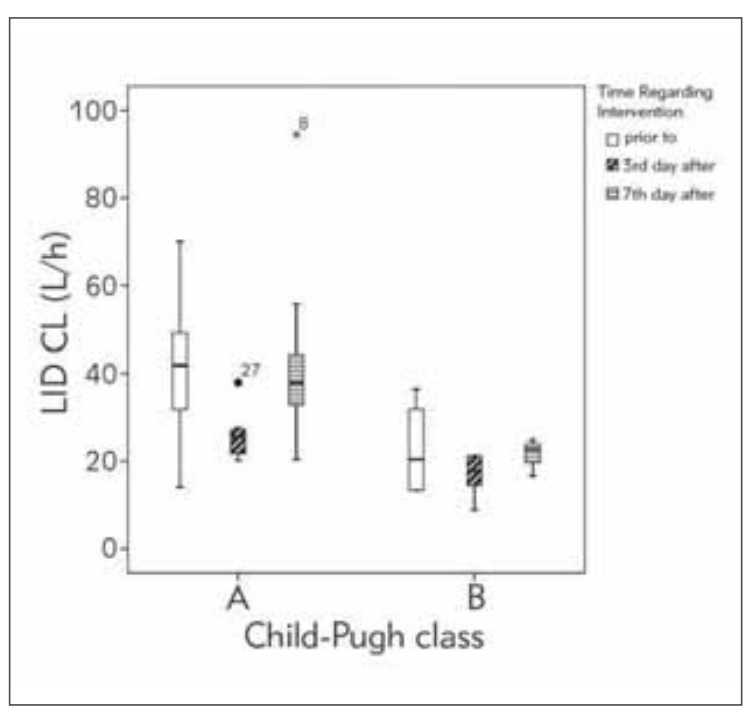
Boxplot of lidocaine (LID) clearance (CL) values with respect to Child-Pugh class in patients with impaired liver function in relation to time regarding intervention.

Figure 3 shows the changes in LID CL values in patients with tumors according to the time after liver tumor resection compared to the preoperative value. These results clearly indicate worsening hepatic function on 3^rd^ day after operation in comparison to the values of LID CL prior to operation (mean LID CL for patients with Child-Pugh class A are 25.91 L/h, 41.59 L/h, respectively; while for B class are 16.89 L/h, 22.65 L/h, respectively). However, on day 7^th^, the values of LID CL (mean value for patients with Child-Pugh class A and B are 40.98 L/h and 21.46 L/h, respectively) are increased in comparison to 3^rd^ day after.

## Discussion

Assessing overall metabolic hepatic activity in patients with impaired liver function is vital as it may affect how drugs are being handled by the liver. As already known, there is no optimal marker or probe substance for accurate hepatic function evaluation. Consequently, variety of tests, clinical signs and symptoms, and diagnosis are considered in order to estimate hepatic function in clinical practice [3] [5]. LID is used as test substance in our study in order to assess its pharmacokinetic characteristics and correlation with Child-Pugh score. Additionally, the analysis aimed in assessing if CL of LID is altered by days in relation to hepatic intervention as liver function may change over time.

The results of our study indicate that LID pharmacokinetic parameters differ from average values in health individuals indicating decrease in LID elimination, determined by CL, due to the liver injury [10]. As given on Figure 2, CL and Vd show greater interindividual variability in relation to t_1/2_. This is expected as CL and Vd are primary pharmacokinetic parameters that reflect physiological and pathophysiological characteristics of patients, while t_1/2_ is secondary parameter, and does not fully and individually represent the patient's status of LID pharmacokinetics. The value of t_1/2_ is calculated from CL and Vd. Various factors can affect LID Vd such as fluid balance including a presence of ascites and its degree, impaired level of proteins and protein/tissue binding, while LID CL is dependent on blood flow excluding protein level, and intrinsic clearance [3] [25]. Figure 2 suggests that there are differences in the metabolic function of liver in relation to the pathophysiological status of liver presented as different diagnosis. LID CL was lower in cirrhotic patients in comparison to tumor, in both Child-Pugh classes. Some studies found that LID extraction ratio and LID CL in decompensated cirrhotic patients were no longer related to liver blood flow but rather became capacity-limited [26].

It is possible to observe great interindividual variability in pharmacokinetic parameters within one Child-Pugh class as given on Figure 2 and Figure 3. This might be due to the fact that this classification system does not adequately represent how drug is being handled in the body and its metabolic activity, but disease outcome prediction [3] [27]. In view of the fact that liver function is not static but it changes with time, we observed changes in LID CL values in patients with tumors according to the time period regarding the operation as presented on Figure 3. These results clearly indicate worsening hepatic function on 3^rd^ day after operation in comparison to the values of LID CL prior to operation (mean LID CL for patients with Child-Pugh class A are 25.91 L/h, 41.59 L/h, respectively; while for B class are 16.89 L/h, 22.65 L/h, respectively). However, on day 7^th^, the values of LID CL (mean value for patients with Child-Pugh class A and B are 40.98 L/h and 21.46 L/h, respectively) are increased in comparison to 3^rd^ day after. Relying only on Child-Pugh class, it would not be possible to observe these negligible changes in liver function. Hence, indocyanine and LID/MEGX test were recommended for assessing liver function in critically ill patients [2]. Mean values of LID CL on 3^rd^ day post intervention are the indicators of the so-called »metabolic storm« which indicated that the organ was in the specific condition, and was still not adapted to its function [28] [29]. In liver cancer patients undergoing laparoscopic hepatectomy, observed prolonged metabolism of LID and MEGX might be related to the hepatic blood flow occlusion or liver injury caused by hepatectomy [30]. Additionally, the resection of the liver parenchyma resulted in the reduced volume and mass of the liver tissue [31] [32]. Therefore, the decrease in LID CL could be expected soon after the surgical intervention, which was measured on 3^rd^ day. Furthermore, it was shown that anesthesia and surgery may deteriorate liver function in patients undergoing non-hepatic surgery [33]. In our study, LID CL returns to almost preoperative values on 7^th^ day, which means that the liver parenchyma and metabolic function recover within a week after the resection.

The presented results of the study confirmed that the mean value of LID CL decreases, and t_1/2_ increases with the Child-Pugh class as similar results were showed in previous studies [21] [22] [34]. Results of our study confirm importance of LID pharmacokinetic parameters as previously published [21] [22]. It should be highlighted that in the study by Munoz et al. [22] LID was administered *per os*, thus our study is the first one which gives the values of LID pharmacokinetic parameters after *i.v.* administration in the patients with liver impairments. The presented results show that LID CL is in better correlation with Child-Pugh class over t_1/2_, and Vd. The results show great interindividual variability in pharmacokinetic parameters of LID within one group determined by Child-Pugh classification.

The main limitation of this study is the absence of healthy control group, and consequently results were compared with previous findings. Nevertheless, comparison between patients with cirrhosis and liver tumor, as well as between different stages of liver impairment represented by Child-Pugh class, enables deeper insight and understanding of LID pharmacokinetics dependence on the type and progression of disease, which implies its prognostic value. Although discrepancy in average age between patients with cirrhosis and tumor was observed, elimination of highly metabolized drug is primarily determined by the liver status. In addition, it would be useful to further assess correlation of LID pharmacokinetic parameters with Model for End-Stage Liver Disease (MELD) score, since it is widely used in clinical practice and overcomes some limitations of Child-Pugh staging system [1].

LID, as well as other probe substances, has disadvantages, and its CL values may be informative since LID eliminates mainly via liver. Based on the results, it is possible to conclude that LID pharmacokinetics, presented by parameters, can be used to asses progression of liver impairment. Consequently, the values of LID CL and Vd calculated after this dynamic test, should be coupled with standard biochemical parameters and clinical assessment, in order to estimate liver function and obtain the complete picture of hepatic status in patients in surgical intensive care units, especially for longitudinal patient's monitoring.

## Dodatak

### Acknowledgements

This research was funded by the Ministry of Education, Science and Technological Development, Republic of Serbia through Grant Agreement with University of Belgrade-Faculty of Pharmacy No. [451-03-68/2022-14/200161]. Moreover, the authors thank Abbott^® ^for the kind donation of the analysis kit to the Clinical Centre of Serbia. 

### Conflict of interest statement

All the authors declare that they have no conflict of interest in this work.

### List of abbreviations

LID, lidocaine;<br>MEGX, monoethyl-glycinexylidide;<br>CL, clearance;<br>Vd, volume of distribution;<br>lz, terminal phase elimination rate constant;<br>t1/2, half-life
